# Dual-Peak Lorentzian CEST MRI for antiretroviral drug brain distribution

**DOI:** 10.1515/nipt-2022-0012

**Published:** 2023-03-25

**Authors:** Yutong Liu, Gabriel C. Gauthier, Howard E. Gendelman, Aditya N. Bade

**Affiliations:** Department of Pharmacology and Experimental Neuroscience, University of Nebraska Medical Center, Omaha, NE, USA; Department of Radiology, University of Nebraska Medical Center, Omaha, NE, USA

**Keywords:** antiretroviral drug, chemical exchange saturation transfer, lamivudine, magnetic resonance imaging

## Abstract

**Objectives:**

Spatial–temporal biodistribution of antiretroviral drugs (ARVs) can now be achieved using MRI by utilizing chemical exchange saturation transfer (CEST) contrasts. However, the presence of biomolecules in tissue limits the specificity of current CEST methods. To overcome this limitation, a Lorentzian line-shape fitting algorithm was developed that simultaneously fits CEST peaks of ARV protons on its Z-spectrum.

**Case presentation:**

This algorithm was tested on the common first line ARV, lamivudine (3TC), that has two peaks resulting from amino (–NH_2_) and hydroxyl (–OH) protons in 3TC. The developed dual-peak Lorentzian function fitted these two peaks simultaneously, and used the ratio of –NH_2_ and –OH CEST contrasts as a constraint parameter to measure 3TC presence in brains of drug-treated mice. 3TC biodistribution calculated using the new algorithm was compared against actual drug levels measured using UPLC-MS/MS. In comparison to the method that employs the –NH_2_ CEST peak only, the dual-peak Lorentzian fitting algorithm showed stronger correlation with brain tissue 3TC levels, signifying estimation of actual drug levels.

**Conclusions:**

We concluded that 3TC levels can be extracted from confounding CEST effects of tissue biomolecules resulting in improved specificity for drug mapping. This algorithm can be expanded to measure a variety of ARVs using CEST MRI.

## Introduction

Chemical exchange saturation transfer (CEST) contrast arises when an exchangeable proton of a molecule is magnetically saturated then transferred to water by chemical exchange. This incites a reduction in the magnetic resonance imaging (MRI) water signal, reflecting the molecule’s concentration. Continuous proton exchange leads to the buildup of water signal reductions enabling detection of the molecule in tissue. Unlike traditional drug imaging, which requires exogenous imaging contrast agents, CEST effect is an endogenous property of a molecule [1–10]. Indeed, we previously reported CEST effectiveness in detecting brain lamivudine (3TC) biodistribution [11]. The current study serves to advance CEST approach by ameliorating specificity and accuracy of determining antiretroviral drug (ARV) brain tissue biodistribution (BD).

CEST-based data analysis strategies have been examined in previous studies, such as the calculation of symmetric magnetization transfer ratio (*MTRasym*) from a Z-spectrum [12–16]. A Z-spectrum is the plot of water MRI signals at varying saturation frequencies. *MTRasym* is calculated by subtracting downfield water signals (at offset frequencies) from upfield water signals (at frequencies lower than water). However, *MTRasym* is confounded by magnetization transfer contrasts (MTC) from semi-solid macromolecules along with relayed nuclear Overhauser effects (rNOE) of macromolecules. To improve CEST specificity, a variety of quantification methods have been developed that included Lorentzian line-shape fitting [17–20], AREX [21–23] and chemical exchange rotation transfer [24–26]. Each method is able to differentiate MTC, rNOE and water spillover that is also called as water direct saturation (DS). Our previous works used 5-pool Lorentzian fitting to measure CEST effect of amino (–NH_2_) protons of 3TC to detect drug biodistribution [11]. Nonetheless, some tissue metabolites such as creatine and glutamine can, also, generate CEST peaks at about the same saturation frequency offset (2 ppm) as the 3TC–NH_2_ proton [19, 27], [28], [29], [30], [31], [32]. Therefore, the 2 ppm peak reflects the combined CEST effects of 3TC, creatine, glutamine and other biomolecules that have amine or amino protons. To improve the specificity of 3TC detections, we proposed a dual-peak Lorentzian line-shape function. This fitting function is used to account for the CEST peaks of –NH_2_ and –OH protons simultaneously. The inclusion of the –OH CEST effect adds a constraint for the fitting of –NH_2_ proton, and thus improves 3TC detection specificity. The proposed algorithm uses a 2-step fitting strategy to fit the *in vivo* Z-spectrum. In this approach background signal from direct saturation, MTC, and other biomolecules is fitted using a polynomial function. The peaks of –NH_2_ and –OH protons are then fitted using a dual-peak Lorentzian function. The method is now validated against drug levels measured using ultra performance liquid chromatography – tandem mass spectrometer (UPLC-MS/MS).

## Materials and methods

### CEST MRI of 3TC-treated mice

All animal studies were approved by the University of Nebraska Medical Center Institutional Animal Care and Use Committee (IACUC) in accordance with the standards incorporated in the Guide for the Care and Use of Laboratory Animals (National Research Council of the National Academies, 2011). Complete details about animal administration with 3TC and MRI acquisitions were previously described [11]. Briefly, male C57BL/6 mice (14–16 weeks old) were treated by oral gavage for five days with 3TC (250 mg/kg) or vehicle (0.2% hydroxypropylmethyl cellulose and 0.1% Tween 80 in sterile water; control group). CEST MRI was performed on a 7 Tesla MRI scanner (Bruker BioSpec 70/20, Billerica, MA). Experiments were performed using a RARE sequence (TR/TE = 1600/16 ms, RARE factor = 8) with a continuous RF for saturation with the power = 2 µT, duration = 1 s, saturation frequencies = −5 to 5 ppm in steps of 0.2 ppm. A library of prior CEST data sets were used for comparison with the dual-peak Lorentzian fitting algorithm.

### Theory

**Figure 1:**
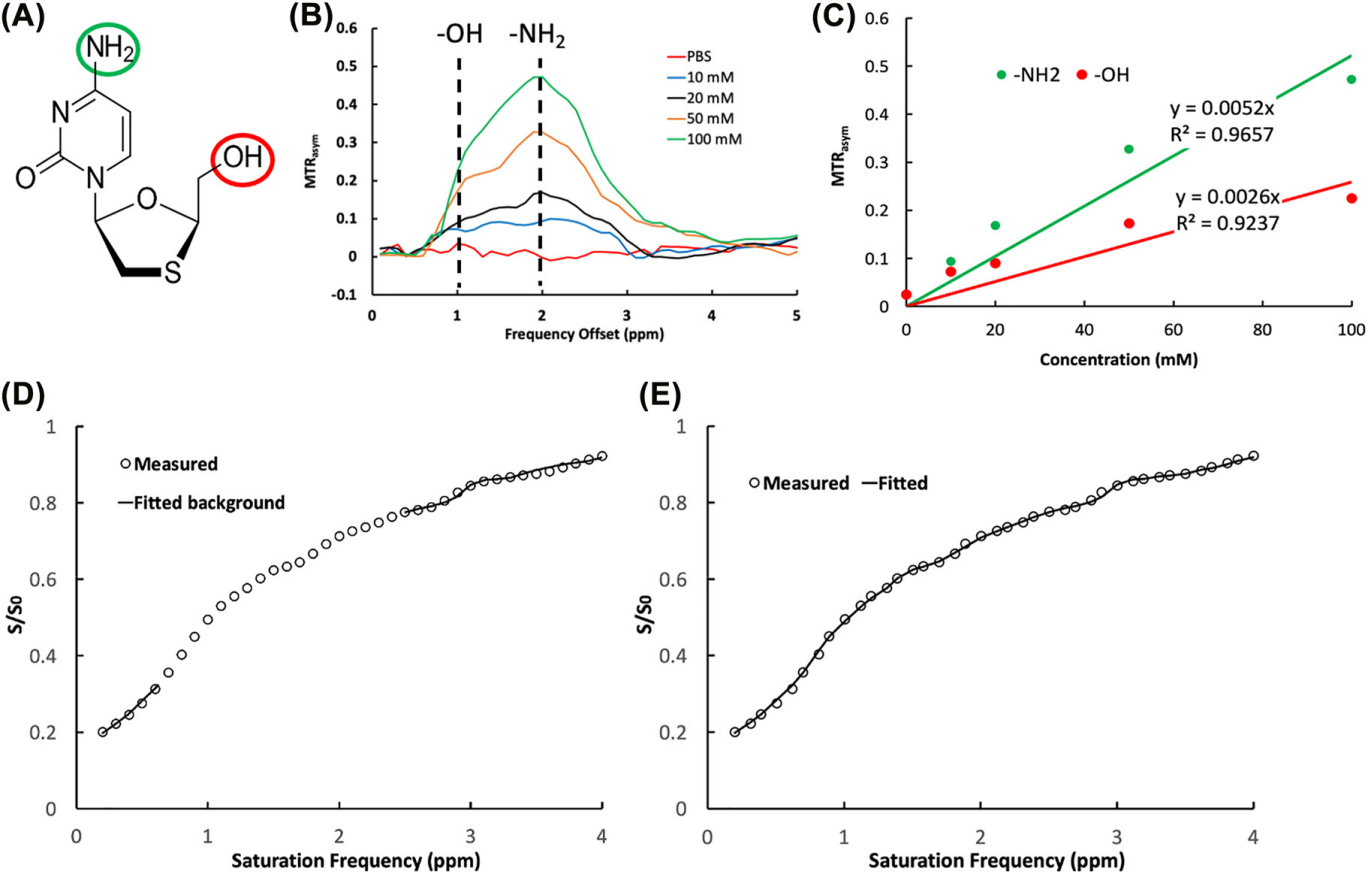
Dual-peak Lorentzian fitting based on the CEST effects of -NH_2_ and -OH in 3TC. (A) Lamivudine (3TC) chemical structure. (B) MTRasym calculated from Z-spectra of 3TC. The CEST effects of –NH_2_ and –OH protons are at 2 and 1 ppm, respectively. MTRasym results are extracted from a previous study [11] and adapted for re-analysis. (C) CEST effects of –NH_2_ and –OH protons are proportional to 3TC concentration (R^2^=0.97 and 0.92 for –NH_2_ and –OH, respectively). CESTivity (line of slope) is 0.0052 mM^−1^ for –NH_2_ proton of 3TC and 0.0026 for –OH proton of 3TC. (D) Background fitting using a polynomial function. (E) A dual-peak Lorentzian function fits –NH_2_ and –OH.

A dual-peak Lorentzian fitting method was deployed to simultaneously analyze CEST effects of –NH_2_ and –OH protons of 3TC (Figure 1A). The 3TC CEST *MTRasym* analyses are illustrated in Figure 1B. The CEST effects of –NH_2_ and –OH protons were proportional to 3TC concentrations with correlation coefficients for –NH_2_ and –OH protons at *R*^2^=0.97 and 0.92, respectively (Figure 1C). Increasing rates of CEST contrasts with 3TC concentrations (slope of the line) were 0.0052 mM^−1^ for –NH_2_ proton, and 0.0026 mM^−1^ for –OH proton, respectively. The slope was defined as “CEST*ivity*” reflecting the CEST effect change of a proton with drug concentration. The CEST*ivity* of –NH_2_ proton was twice that of the –OH proton (Figure 1D). Dual-peak Lorentzian functions were developed to fit the CEST effects of –NH_2_ and –OH protons. This was achieved by converting Z-spectrum data into a linear combination of water relaxation rates of the –NH_2_ and –OH protons in the rotating frame under RF saturation by

(1) $$Z\left(\%\right)=\frac{\cos^{2}\theta R_{1}}{R_{1\rho }},$$

where *Z* (%) is the normalized Z-spectrum that is the original Z-spectrum normalized by unsaturated water signal, *R*_1_ is the longitudinal relaxation rate of water, *θ* is the tilt angle of the effective magnetization with respect to *Z*-axis, and *R*_*Iρ*_ is the water relaxation rate [29–36]. For animal data,

(2) $$R_{1\rho }=R_{bg}+R_{\text{NH}2}+R_{OH},$$

where *R*_NH2_ and *R*_OH_ are the longitudinal relaxation rates of –NH_2_ and –OH protons in the rotating frame, respectively. *R*_*bg*_ accounts for the rotating frame rate of water and all other effects including MTC of semi-solid macromolecules and CEST effects of biomolecules other than 3TC. *R*_*bg*_ is represented by a polynomial function for DS and MTC, and a Lorentzian function for the CEST effect at 3 ppm from the amine and amide protons in biomolecules such as glutamate and mobile proteins:

(3) $$\begin{array}{ll} R_{bg} & =C_{0}+C_{1}\cdot \Delta \omega +C_{2}\cdot \Delta \omega^{2}\\ & \;+R_{3ppm}^{\max}\;\cdot \frac{w_{3ppm}^{2}}{w_{3ppm}^{2}+4{\left(\Delta \omega -\Delta \omega_{3ppm}\right)}^{2}}, \end{array}$$

where *C*_0,_ *C*_1_ and *C*_2_ are the 0th to the 2nd order polynomial coefficients, Δ*ω* is the frequency offset relative to water at 0 ppm $R_{3\;ppm}^{\max}\;$ refers to the apparent relaxation rate of the 3 ppm component, *w*_3ppm_ is the full width at half maximum (FWHM) of the 3 ppm Lorentzian curve, and Δω_3ppm_ is the chemical shift. *R*_NH2_ and *R*_OH_ are fitted using Lorentzian functions:

(4) $$R_{\text{NH}2}=R_{\text{NH}2}^{\max}\;\cdot \frac{w_{\text{NH}2}^{2}}{w_{\text{NH}2}^{2}+4{\left(\Delta \omega -\Delta \omega_{\text{NH}2}\right)}^{2}},$$

and

(5) $$R_{OH}=R_{OH}^{\max}\;\cdot \frac{w_{OH}^{2}}{w_{OH}^{2}+4{\left(\Delta \omega -\Delta \omega_{OH}\right)}^{2}},$$

where *w*_NH2_ and *w*_OH_ are the FWHM of the peaks of amino and hydroxyl Lorentzian curves, respectively. Δω_NH2_ and Δω_OH_ are the chemical shifts of –NH_2_ and –OH protons, respectively. $R_{\text{NH}2}^{\max}\;$ and $R_{OH}^{\max}\;$ refer to the relaxation rates of –NH_2_ and –OH protons, respectively, and they are linked by

(6) $$R_{OH}^{\max}=r_{a/h}\cdot R_{\text{NH}2}^{\max},$$

where the initial value of *r*_*a/h*_ is set to 0.5. This is estimated from the CEST*ivity* ratio of –NH_2_ and –OH protons, which is 2 (Figure 1C) using Eq. (1). The background (Eq. (3)) is first fitted on 0.2–5 ppm that excluded data points between 0.5 and 2.5 ppm. In the second step, the data points in 0.5–2.5 ppm are fitted using Eqs. (4) and (5). The algorithm was first tested on averaged Z-spectra across brain image pixels of mice administered with 3TC (Figure 1D and E), in which the *in vivo* data in the frequency offset range 0.2–5 ppm are shown as dots. The background estimated using Eq. (4) is shown in a solid line in Figure 1D, and the solid line in Figure 1E represents the fitting result of the –NH_2_ and –OH effects using Eqs. (4) and (5). The average goodness-of-fit is higher than 0.9 for all data sets. The inclusion of hydroxyl effect adds a constraint for the fitting of amino proton via *r*_*a/h*_, and thus improves the accuracy of the fitting result. The algorithm is implemented in Matlab (MathWorks, Natick, MA) using “lsqcurvefit” function for both polynomial and Lorentzian fittings.

### Statistical Analysis

Statistical analysis was performed using Matlab. Data were presented as mean ± standard error of the mean (SEM). For comparisons of two groups, Student’s t-tests (one-tailed) were used. Significant differences were determined at p<0.05, and trends of changes were defined as p<0.1. The associations of MRI CEST results and drug levels determined by UPLC-MS/MS were evaluated using Pearson correlations in Matlab.

## Results

### 3TC-treated mice

**Figure 2:**
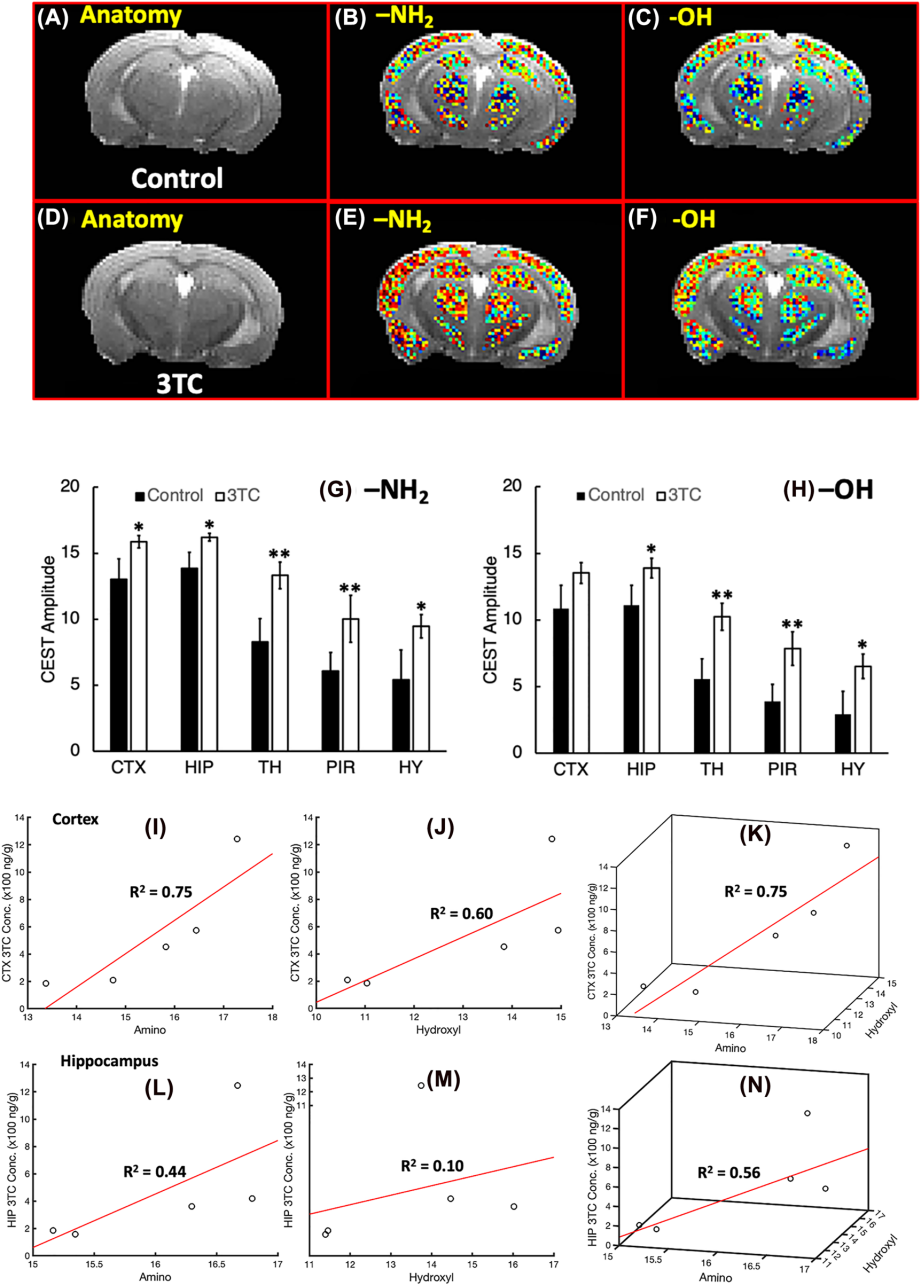
*In vivo* CEST analysis of 3TC-administarted mice. (A)–(C) Anatomical reference image, –NH_2_ CEST amplitude map, and –OH CEST amplitude map of a control mouse. (D)–(F) Anatomical reference image, –NH_2_ CEST amplitude map, and –OH CEST amplitude map of a 3TC-administrated mouse. (G) and (H) Comparisons of CEST effects OF –NH_2_ and –OH protons in cortex (CTX), hippocampus (HIP), thalamus (TH), piriform cortex (PIR) and hypothalamus (HY) using Student’s t-tests. **: p<0.05, *: p<0.1. (I)–(K) Correlations of –NH_2_ (I), –OH (J) and combined CEST effect (K) with drug levels in cortex measured using Pearson’s tests. (L)–(N) correlations of –NH_2_ (I), –OH (J) and combined CEST effect (K) with drug levels in hippocampus measured using Pearson’s tests.

The dual-peak fitting algorithm was tested on CEST MRI data previously acquired [11]. CEST effects of 3TC dosing were measured on brain sub-regions of C57BL/6 male mice. 3TC was administered orally for 5 days (250 mg/kg/day), in accordance to animal equivalent dose (AED) conversions [37], with dosing matched to the equivalent of five times the human dose. Control animals received 0.2% hydroxypropylmethyl cellulose and 0.1% Tween 80 in sterile water as a vehicle by equivalent administration routes. T_2_-weighted images were used as an anatomical reference for brain region-of-interest (ROI) evaluations (Figure 2A and D). CEST 3TC effects were measured in five brain sub-regions, including the hippocampus (HIP), cortex (CTX), piriform cortex (PIR), thalamus (TH) and hypothalamus (HY). The CEST maps on brain regions of 3TC and vehicle-treated mice are shown in Figures 2A–F. Stronger CEST effects of –NH_2_ and –OH protons were noted in the brain of drug treated mice (Figure 2E and F) compared to the vehicle-treated mice (Figure 2B and C). CEST effect of –NH_2_ proton was significantly higher in 3TC-treated mice (n=7) compared to controls (n=8) on thalamus (p=0.022) and piriform cortex (p=0.049) (Figure 2G). The trend of significant increase was observed on brain regions, cortex (p=0.070), hippocampus (p=0.059) and hypothalamus (p=0.078) in drug-treated animals. The CEST effect of –OH proton was higher in the thalamus (p=0.018) and piriform cortex (p=0.029) in 3TC-treated mice. Trends of significant increase were noted for –OH proton effects on hippocampus (p=0.076) and hypothalamus (p=0.058) in the drug treated group.

Next, we correlated CEST MRI results obtained by the dual-peak fitting algorithm with drug levels measured using UPLC-MS/MS in the 3TC-administered group (n=5) in the cortex and hippocampus regions (Figure 2I–N). In the cortex, –NH_2_ and –OH CEST effects were correlated with drug levels at coefficients *R*^2^=0.75 and 0.60, respectively (Figure 2I and J), whereas the correlation coefficient of the combined CEST effects of –NH_2_ and –OH protons with drug levels was *R*^2^=0.75 (Figure 2K). Within the hippocampus, the correlation coefficients of drug levels with –NH_2_ proton and with –OH proton were *R*^2^=0.44, and *R*^2^=0.10, respectively, (Figure 2L–M). Combined CEST effects of –NH_2_ and –OH protons with measured tissue drug levels showed a correlation coefficient of *R*^*2*^=0.56 (Figure 2N).

### –NH_2_ proton -based algorithms

Results from the dual-peak fitting algorithm for 3TC biodistribution in brain regions were compared to results from a prior algorithm based only on –NH_2_ proton- CEST effect (Table 1). In the –NH_2_-only-based algorithm, the correlation coefficient of CEST effect in the cortex with drug levels was *R*^2^=0.62 (Table 1) [11]. Whereas, the constrained –NH_2_ CEST effects with the new algorithm showed higher correlation with drug levels, *R*^2^=0.75 (Figure 2, Table 1). These data demonstrated a high accuracy of dual-peak fitting algorithm for drug detection. The correlation coefficient of drug levels with combined –NH_2_ and –OH proton CEST effects was similar to that of constrained –NH_2_ (*R*^2^=0.75). In hippocampus region, accuracy of drug detection was improved after employing the dual-peak fitting algorithm. The correlation of constrained –NH_2_ effect with drug levels was *R*^2^=0.44 compared to *R*^2^=0.20 by the prior algorithm. Also, the correlation coefficient of combined –NH_2_ and –OH effects showed higher correlation coefficient *R*^2^=0.56. Taken together, the –NH_2_ CEST effect fitted with constraints correlated closely to actual drug levels. The correlation was further improved when the combined CEST effect of constrained –NH_2_ and –OH was deployed.

**Table 1:** Correlation coefficients between brain region 3TC and CEST.

	–NH_2_ only	Constrained –NH_2_	Constrained –OH	Combined –NH_2_ and –OH
CTX	0.62^a^	0.75	0.60	0.75
HIP	0.20^a^	0.44	0.10	0.56

^a^From [11].

## Discussion

In traditional drug imaging modalities, drug molecules are tagged with imaging contrast agents or loaded with imaging contrast agents into nanoparticles. Usually paramagnetic metals are used for MRI (2–4) and radioactive materials for PET and SPECT (2–8) tests. Similar techniques were deployed to assay the biodistribution of nanoformulated ARVs [38–41]. The limitations of the prior methods are significant [1–10]. *First*, the loading rates of nanoparticles are usually limited to achieve effective therapy and/or imaging sensitivity [42]. *Second*, toxicity can be associated with imaging agents and nanoparticles [43–45]. *Third*, blood-brain barrier (BBB) penetration needs to be considered when designing traditional drug imaging methods for the CNS. Previously, we showed that the CEST effect can be used for *in vivo* imaging of 3TC [11]. Unlike traditional methods, the CEST contrast comes as a consequence of the drug’s chemical structure and therefore does not need extrinsic imaging agents. Thus, CEST imaging eliminates the limitations associated with extrinsic imaging agents used in traditional techniques. In this study, we developed the analysis algorithm to improve the accuracy and specificity of *in vivo* CEST MRI mapping of ARVs.

The 2-step fitting strategy, in which fitting of the background Z-spectrum is followed by the fitting of CEST peaks of interest, was used in previous studies for detection of creatine and phosphocreatine [29, 30, 32, 46]. The innovation of our algorithm is the simultaneous fitting of peaks of –NH_2_ and –OH protons that are linked and constrained via the CEST*ivity* ratio (*r*_*a/h*_) in Eq. (6). This extracts the –NH_2_ effect of 3TC from the peak that can contain effects from other tissue biomolecules such as creatine and glutamine serving to improves the specificity of 3TC detection. In summary, we successfully developed a new algorithm that uses the CEST effects of both amino (–NH_2_) and hydroxyl (–OH) protons for *in vivo* 3TC detection. The new algorithm shows high specificity for 3TC biodistribution measurements which can be extended to other ARVs.
